# Immune cell crosstalk between ANCA-associated vasculitis and IgG4-related disease: an unresolved pathogenic link

**DOI:** 10.3389/fimmu.2025.1660956

**Published:** 2025-10-16

**Authors:** Cui Wang, Ronghua He, Xue Bai, Yarui Zhang, Jiawen Li, Jie Zhao, Wenhui Gao, Qiaoyan Guo

**Affiliations:** ^1^ Department of Nephrology and Rheumatology, The Second Hospital of Jilin University, Changchun, Jilin, China; ^2^ Department of General Practice, The First Hospital of Jilin University, Changchun, Jilin, China; ^3^ Department of Neonatology, The Second Hospital of Jilin University, Changchun, Jilin, China

**Keywords:** IgG4-RD, AAVs, GPA/MPA, pathophysiology, overlap syndrome

## Abstract

Immunoglobulin G4-related disease (IgG4-RD) is a rare, multisystemic fibro-inflammatory condition affecting various organs, including kidneys, lungs, nasal cavity, pancreas, salivary glands, and orbit. Anti-neutrophil cytoplasmic antibody (ANCA)-associated vasculitis (AAVs) is a multi-systemic inflammatory vascular disease encompassing eosinophilic granulomatosis with polyangiitis (EGPA), microscopic polyangiitis (MPA), and granulomatosis with polyangiitis (GPA). It often overlaps with the organs or tissues affected by IgG4-RD. Clinically, some individuals with IgG4-RD are ANCA-positive, while some with AAV exhibit elevated IgG4 levels or IgG4-positive plasma cell infiltration, making these conditions difficult to distinguish. Reports have documented cases of overlap syndromes involving IgG4-RD and AAV, highlighting shared pathogenic mechanisms that may include macrophages, B cells, CD4+T cells, and inflammatory cytokines. However, the pathophysiological mechanism underlying these overlap syndromes remains unclear. This review examines potential pathophysiological links between IgG4-RD and AAVs (GPA/MPA) overlap syndromes.

## 1 Introduction

IgG4-RD is a rare fibroinflammatory condition characterized by the infiltration of IgG4-positive plasma cells, tumor-like mass formation, and elevated serum IgG4 levels (1). It affects a diverse range of organs, including the salivary glands, periorbital tissues, kidneys, lungs, pancreas, nasal cavity, pericardium, and skin (2). Approximately 15% of individuals with IgG4-RD exhibit renal involvement, predominantly tubulointerstitial nephritis (TIN), while a smaller proportion may develop secondary membranous nephropathy (3, 4). The characteristic histological features of IgG4-RD include dense lymphocytic inflammation (IgG4-positive plasma cells > 10 per high-power field or IgG4/IgG ratio > 40%), storiform fibrosis, and obliterative phlebitis (5). ANCA positivity is observed in some IgG4-RD cases (6), raising questions about the potential overlap between IgG4-RD and AAVs (7).

**Figure 1:**
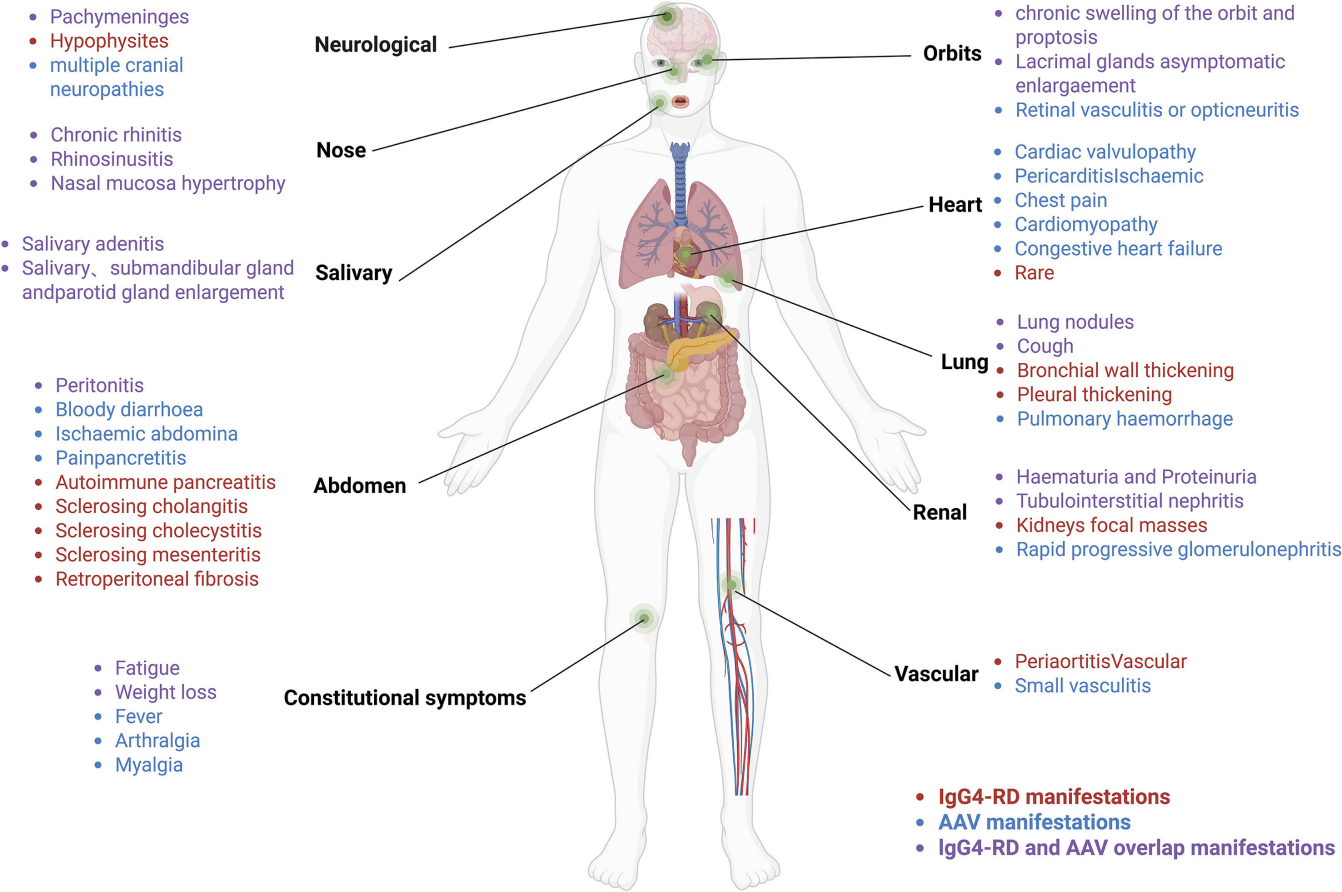
IgG4-RD overlaps with affected organs in MPA/GPA.

AAVs are autoimmune conditions characterized by vascular inflammation, endothelial damage, and tissue injury, often involving kidneys, lungs, sinuses, periorbital tissues, and salivary glands (8), sites that frequently overlap with those affected in IgG4-RD (9) (**Figure 1**). AAVs are clinically classified into three subtypes: GPA, MPA, and EGPA (10). Over 75% of individuals with AAVs experience renal involvement, often manifesting as rapidly progressive glomerulonephritis, including hematuria, proteinuria, and reduced glomerular filtration rate (11). ANCAs are common biomarkers for AAVs, typically IgG, with IgG4-ANCA being the predominant subtype when MPA overlaps with IgG4-RD (12). Proteinase 3 (PR3) and myeloperoxidase (MPO) are the main target antigens of ANCAs (13). Approximately 60% of individuals with MPA are MPO-ANCA positive, exhibiting features such as necrotizing glomerulonephritis and pulmonary vasculitis (14), typically without granulomatous inflammation (15). Some individuals with MPA present with atypical symptoms, including pachymeningitis, orbital swelling, or chronic periaortic inflammation (9, 16), which may indicate overlap with IgG4-RD. GPA is predominantly PR3-ANCA positive in approximately 75% of cases and is commonly characterized by upper respiratory tract inflammation, pulmonary hemorrhage, granulomatous inflammation, and glomerulonephritis (17). Notably, some GPA cases exhibit IgG4-positive plasma cell infiltration, infiltration on biopsies of the head and neck, such as sinuses and periorbital region, mimicking IgG4-RD (18). EGPA, while less prevalent than GPA and MPA, is frequently MPO-ANCA positive and primarily manifests as asthma, eosinophilia, and vasculitis (19). It demonstrates unique genetic, pathogenetic, and clinical features, distinguishing it as a separate entity (20, 21). Thus, this discussion focuses primarily on the pathogenesis of MPA/GPA.

Both IgG4-RD and AAVs are autoimmune diseases with notable similarities in organ involvement, clinical presentation, serology, imaging, and histopathology (18, 22, 23). Reports of IgG4-RD overlapping with MPA/GPA are increasing (13, 16, 24–27) (See **Supplementary Table 1**), suggesting the emergence of a novel overlap syndrome (9). This overlap implies potential pathophysiological connections between these conditions. Despite shared features such as B-cell maturation, CD4+T-cell differentiation, macrophage activation, and cytokine secretion, the pathophysiological mechanisms linking IgG4-RD and AAV overlap syndrome remain unclear. This review explores these potential connections to provide a foundation for improved diagnosis and early intervention in these diseases.

## 2 Immuno-pathophysiological mechanisms in IgG4-RD

**Figure 2:**
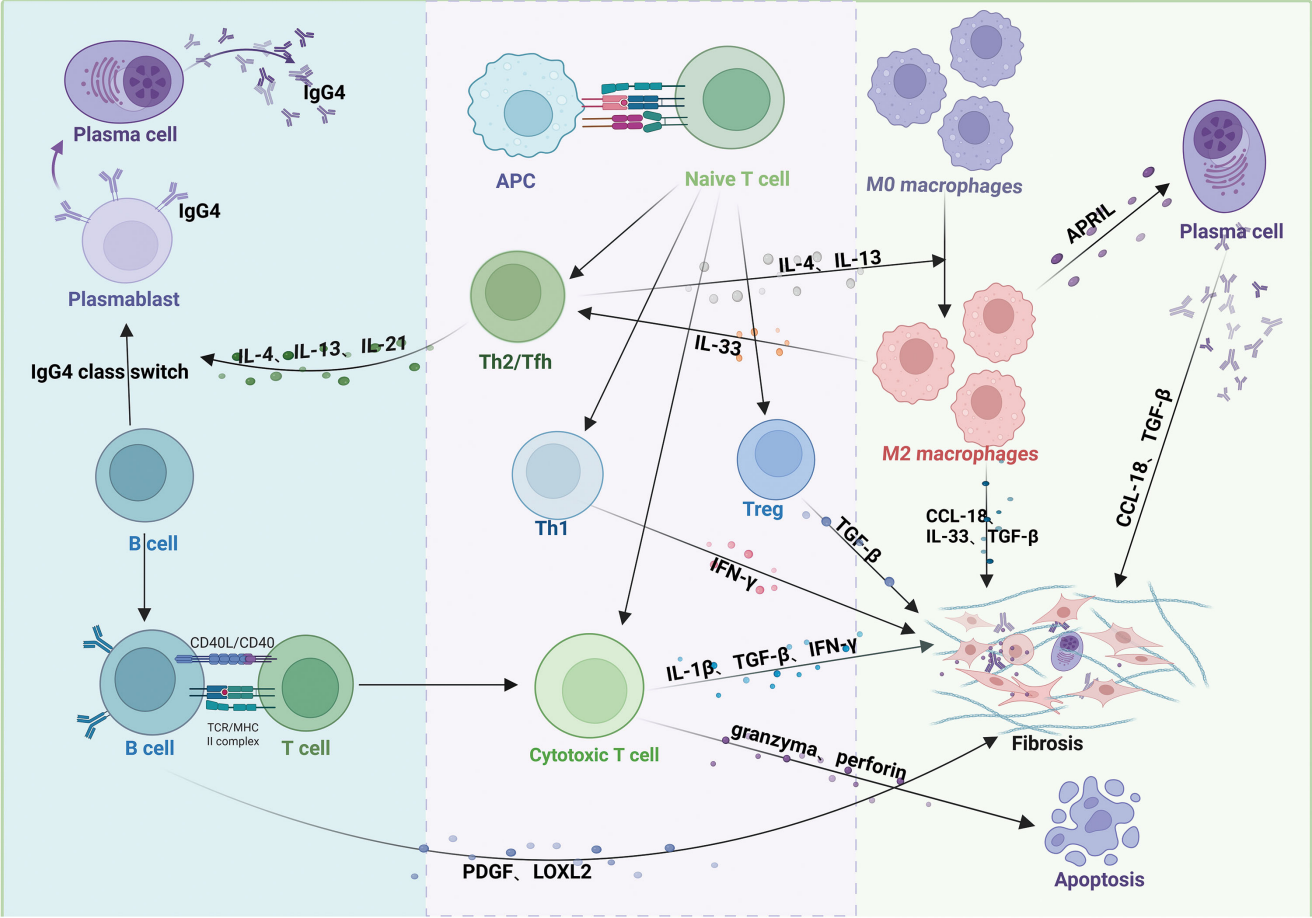
Pathogenic mechanisms of IgG4-related disease (IgG4-RD). Antigen-presenting cells (APCs) activate T cells, promoting differentiation into distinct subsets. Th2 and Tfh cells secrete IL-4, IL-13, and IL-21, driving B-cell maturation into plasma cells or plasmablasts and IgG4 class-switching. Th1 cells secrete IFN-γ; Treg cells secrete TGF-β; and CD4+ cytotoxic T lymphocytes (CTLs) secrete IFN-γ, IL-1β, and TGF-β. These T-cell subsets collectively promote fibrosis in affected tissues. CD4+ CTLs additionally induce apoptosis via granzyme/perforin release. Activated B cells produce PDGF and LOXL2, activating fibroblasts and exacerbating fibrosis. IL-4/IL-13 from Th2 cells polarizes macrophages to an M2 phenotype. M2 macrophages enhance Th2 activation via IL-33 (positive feedback) and, alongside Th1 cells, Tregs, and CD4+ CTLs, contribute to fibrosis through profibrotic mediators (e.g., TGF-β, CCL-18).

In IgG4-RD, antigens activate the innate (e.g., macrophages) and adaptive (e.g., T-lymphocytes and B-lymphocytes) immune systems (28, 29). Extensive infiltration of these immune cells leads to organ swelling, storiform fibrosis, and obliterative phlebitis, as observed in tissue biopsies (30) (**Figure 2**).

B-lymphocytes play a central role in IgG4-RD pathogenesis, primarily differentiating into IgG4-positive plasma cells and infiltrating affected tissues (31). The clinical symptoms of patients with IgG4-RD significantly improve after depletion of the B cell lineage with rituximab (anti-CD20 monoclonal therapy), demonstrating the important pathogenic role of B cells in IgG4-RD (32, 33). T-cells contribute to B cell proliferation and differentiation in IgG4-RD (34). For example, T helper type 2 (Th2) and T follicular helper (Tfh) cells produce cytokines such as interleukin (IL)-4, IL-21, and IL-13, promoting B-cell maturation into plasma cells or plasmablasts and facilitating IgG4 isotype switching (35), highlighting the T cell-dependent nature of B-cell activation. In contrast, T helper type 1 (Th1) cells secrete interferon-gamma (IFN-γ), which induces tissue fibrosis (36). Activated B cells present antigens to T cells via major histocompatibility complex class II, stimulating CD4+ T cells to differentiate into cytotoxic T lymphocytes (CD4+CTLs) and secrete chemokines such as C-C motif chemokine ligand (CCL)-5, which attract CD4+CTLs to affected tissues in IgG4-RD (37). CD4+CTLs aggregate around fibroblasts and release cytokines, including IL-1β, transforming growth factor-beta (TGF-β), and IFN-γ, which promote tissue fibrosis (30, 38). They also induce apoptosis by releasing granzymes and perforin (39). Additionally, activated B cells produce platelet-derived growth factors (PDGF) and Lysyl oxidase-like 2 (LOXL2), activating fibroblasts or collagen fibers and exacerbating fibrosis in affected tissues (40).

Macrophages, particularly M2 macrophages, also contribute to the pathogenesis of IgG4-RD (41, 42). IL-4 and IL-13, produced by Th2 cells, drive macrophage polarization toward the M2 phenotype (43). In turn, M2 macrophages promote Th2 cell activation through the secretion of cytokines like IL-33 (44). M2 macrophages also produce IL-33, CCL-18, and TGF-β, which cause collagen deposition and extracellular matrix protein accumulation, thereby contributing to tissue fibrosis (42, 45, 46). Furthermore, macrophages express a plasma cell survival factor known as a proliferation-inducing ligand (APRIL), which supports plasma cell infiltration and enhances IgG4 production in IgG4-RD (47).

## 3 Immuno-pathophysiological mechanisms in AAV

**Figure 3:**
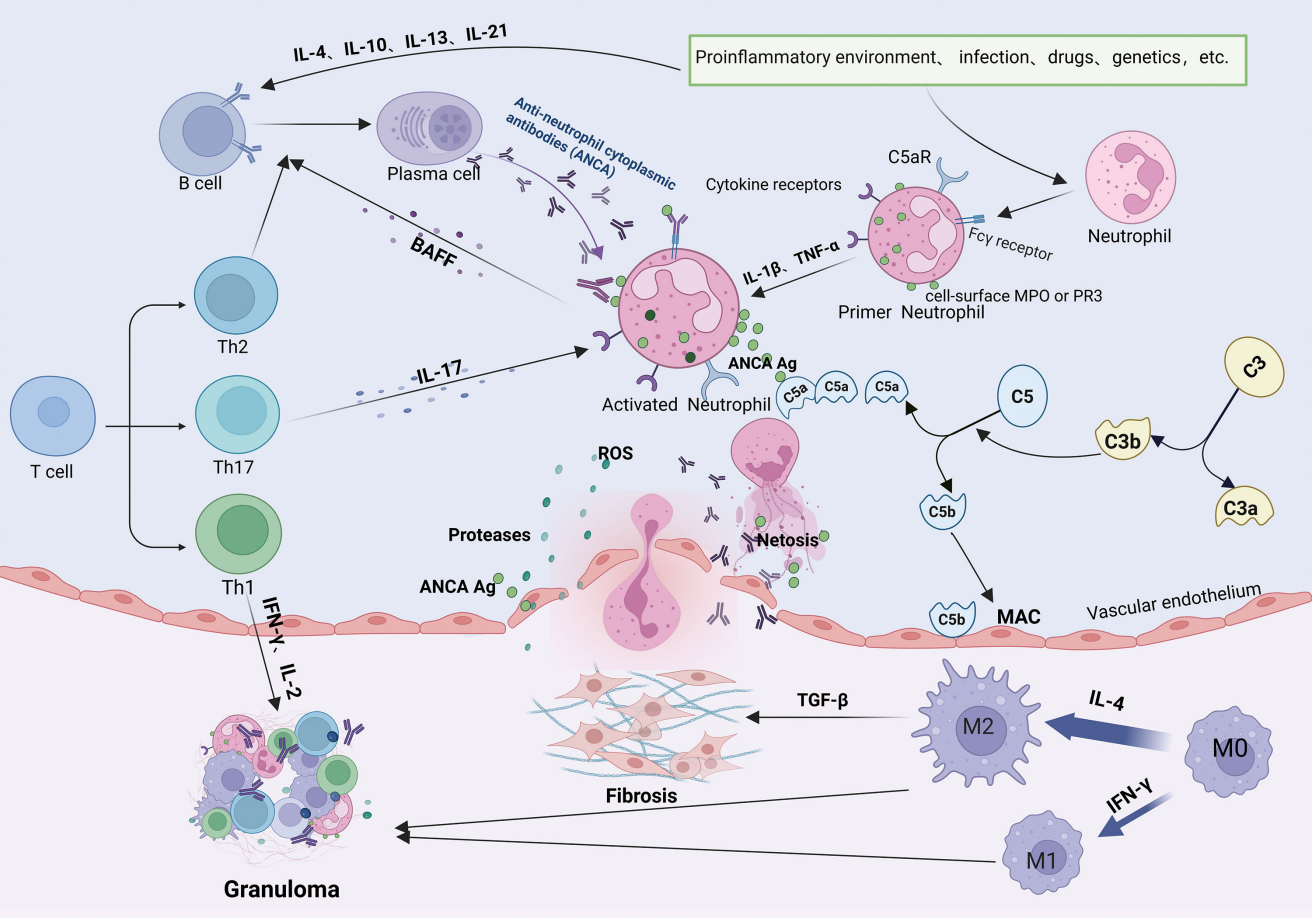
Pathogenic mechanisms of ANCA-associated vasculitis. Loss of B- and T-cell tolerance to ANCA antigens-triggered by inflammation, infections, drugs, or genetic factors-enables B-cell differentiation into antibody-producing plasma cells generating PR3-ANCA or MPO-ANCA antibodies. Antibodies bind to PR3/MPO antigens on neutrophils and synergize with complement (C5a) and cytokines (IL-17) to activate neutrophils. IL-17 further recruits neutrophils to inflammatory sites, where they release ROS, NETs, and proteolytic enzymes, inducing endothelial apoptosis, vascular destruction, and tissue injury. Activated neutrophils secrete BAFF, and Th2/Tfh cells produce IL-4/IL-21, collectively perpetuating pathogenic B-cell responses and autoantibody production. Concurrently, macrophage polarization (M2) and immune cell infiltration drive granuloma formation.

MPA and GPA are characterized by loss of immune tolerance to PR3 and MPO antigens on neutrophils, leading to necrotizing small vessel vasculitis, endothelial damage, and tissue fibrosis (48). B-lymphocytes are important in AAV pathogenesis, maturing and differentiating into plasma cells that produce ANCAs under the influence of cytokines such as IL-4, IL-10, IL-13, and IL-21 (49) (**Figure 3**).

Additionally, B cells present antigens and provide co-stimulatory signals to activate T cells. Activated T cells exacerbate vascular inflammation responses by recognizing neutrophil surface antigens (50). Different subtypes of T cells have distinct roles in MPA/GPA pathogenesis. Th1 cells produce cytokines such as IFN-γ and IL-2, promoting localized inflammatory responses and granuloma formation in GPA (51). Th2 cells secrete IL-4 and IL-13, facilitating plasma cell differentiation and ANCAs production (52). Under inflammatory conditions involving cytokines like IL-6, IL-23, and TGF-β, Th cells can differentiate into T helper type 17 (Th17) cells, which recruit neutrophils to affected tissues through IL-17 production (53). Neutrophils activated by ANCAs, IL-1β, and tumor necrosis factor-alpha (TNF-α) play a central role in MPA/GPA pathogenesis. These neutrophils translocate MPO and PR3 antigens to their surface (54, 55). Neutrophil extracellular traps (NETs) further amplify inflammation by activating complement component C5a, which binds to the C5a receptors on the neutrophil’s surface (56), perpetuating a cycle of activation (57). Activated neutrophils release reactive oxygen species (ROS), proteases, and inflammatory cytokines, damaging vascular endothelial cells and promoting tissue injury (58). ANCAs also stimulate neutrophils to secrete B-cell activating factor (BAFF), enhancing B-cell differentiation and contributing to AAV relapse (59).

In MPA and GPA, biopsies of kidney and lung tissues reveal a significant increase in M2 macrophage infiltration (60, 61), suggesting their involvement in disease progression. MPO-ANCA induces the activation of M2 macrophages and the secretion of TGF-β, thereby exacerbating fibrosis (62). While M2 macrophages can exhibit anti-inflammatory effects through phagocytose apoptotic cells, a process called efferocytosis, PR3 inhibits this process (63), leading to incomplete neutrophil clearance and pro-inflammatory M1 macrophage involvement (64), which participate in GPA granuloma formation together with M2 macrophages (65).

**Figure 4:**
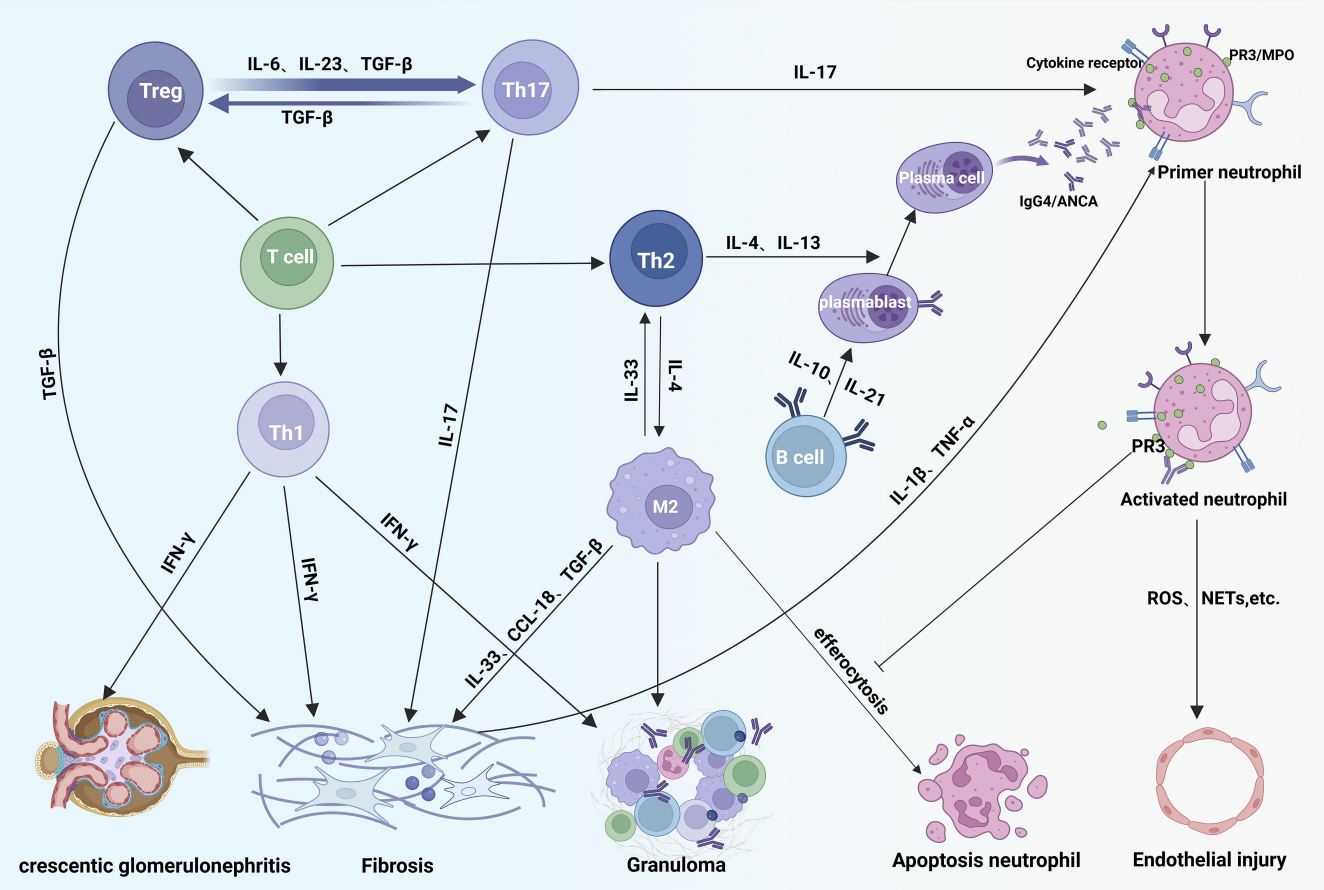
Immunopathogenic overlap in IgG4-RD and ANCA-associated vasculitis. In disease overlap syndromes, Th2-derived IL-4/IL-13 drive B-cell maturation and production of IgG4-class ANCA autoantibodies that bind neutrophil surface receptors, exacerbating vascular inflammation. Concurrently, Th1 cells promote tissue fibrosis and acute crescent formation via IFN-γ. Treg cells differentiate into Th17 cells under IL-23/IL-6/TGF-β stimulation; Th17-secreted IL-17 activates neutrophils, amplifying inflammation. IL-4 from Th2 cells polarizes macrophages toward an M2 phenotype. While M2 macrophages clear apoptotic neutrophils via efferocytosis, membrane PR3 on neutrophils inhibits this process, leading to neutrophil accumulation and sustained M2 activation that intensifies tissue damage. Fibrosis is aggravated by profibrotic mediators: IFN-γ (Th1), TGF-β (Treg), IL-17 (Th17), and IL-10/IL-13/CCL-18 (activated M2 macrophages) acting on fibroblasts.

In summary, the pathogenesis of IgG4-RD and MPA/GPA are complex and multifaceted, involving B cells, T cells, macrophages, and numerous cytokines (e.g., IL-4, IL-13, IL-21, IL-17, TGF-β) (**Table 1**). These shared mechanisms suggest that IgG4-RD and MPA/GPA in overlap syndromes may be pathophysiological linked, potentially creating a feedback loop that worsens both conditions (**Figure 4**).

**Table 1:** The role of various cytokines in IgG4-RD and MPA/GPA.

Cytokines	Secreting cells	IgG4-RD	MPA/GPA	References
APRIL/BAFF	Macrophage, Neutrophil	Activate IgG4-positive plasma cells;	Active autoreactive B cell and promote ANCAs production	(47, 66)
IFN-γ	Th1, CD4+CTLs	Involve in IgG4-RD chronic inflammation and fibrosis	Involve in GPA granuloma formation, promote MPA renal crescent formation and promote M1 macrophage differentiation	(51, 67, 68)
IL-4, IL-13	Th2	Promote B-cell differentiation, plasma cell maturation, and IgG4 antibody class switching; promote M2 macrophage differentiation	Promote B cell differentiation and ANCA production	(69–71)
IL-17	Th17	Participate in chronic inflammation and fibrosis	Induce neutrophil aggregation and promote macrophage activation	(72–75)
IL-10	Macrophage, Treg	Assist IL-4 to reduce IgE and promote IgG4 production	Promote the formation of ANCAs	(76)
IL-21	Tfh	Promote B-cell activation and the generation of germinal centers; promote the proliferation of plasmoblast infiltration	Promote the formation of germinal centers, the maturation of plasma cells and the production of ANCA	(77–79)
TGF-β	Treg, Macrophage, CD4+CTLs	Promote massive infiltration in IgG4-TIN and interstitial fibrosis	Promote fibrosis; Assist with IL-6, IL-23 promotes Th17 cell differentiation tendency	(80, 81)
IL-6	B cell, Monocytes, Macrophage	Pro-inflammatory cytokines, positively correlated with IgG4-RD activity; Aggravating IgG4-RD fibrosis; Promoting Tfh differentiation factor and B cell activation factor production	Pro-inflammatory cytokine involved in inducing differentiation tendencies in Th17 cells; Correlate with glomerular crescent formation in MPA mouse models Involve in GPA granuloma formation.	(82–86)
IL-33, CCL-18	Macrophage	Activate Th2 cells; promote fibrosis;	Engage in GPA granuloma	(87–89)
IL-1β	Th1, CD4+CTLs	Activate collagen cells to promote fibrosis	Activate neutrophils to express anti-inflammatory; promotes fibrosis	(38, 90)

## 4 The role of B cells in IgG4-RD and MPA/GPA overlap syndrome

B cells play a pivotal role in the pathogenesis of IgG4-RD and MPA/GPA (37, 91), primarily by differentiating into plasma cells and secreting antibodies such as IgG4 or ANCAs. ANCA positivity has also been observed in some individuals with IgG4-RD (92), possibly due to non-pathogenic autoreactive B- cell secretion. Consequently, ANCA positivity does not exclude IgG4-RD. It has been shown that serum inflammatory markers, including IgG, IgG1, erythrocyte sedimentation rate, and C-reactive protein, are significantly elevated in ANCA-positive individuals with IgG4-RD. ANCA-positive individuals are more likely to exhibit kidney and lymph node involvement compared to ANCA-negative individuals (6). Thus, ANCAs exacerbate the inflammatory response in IgG4-RD, and the presence of MPA/GPA may further worsen IgG4-RD. ANCAs are pathogenic (19), and ANCA positivity often precedes clinical manifestations of AAV (93). Abbas et al. reported a case of PR3-ANCA-positive IgG4-RD confined to the lungs, which progressed to GPA after 16 months of follow-up (94), suggesting that ANCA-positive IgG4-RD may induce or exacerbate MPA/GPA. ANCAs are predominately of the IgG subtype (21). Holland et al. demonstrated that IgG4 subtypes isolated from ANCA antibodies in patients can activate neutrophils (95). Della-Torre et al. suggested that elevated IgG4 production in IgG4-RD promotes ANCA formation (96). High levels of IgG4 in IgG4-RD may activate neutrophils, increasing the risk of overlap with MPA/GPA and exacerbating the progression of MPA/GPA (96). There are significant increases in IgG4 ANCAs that have been observed in MPA/GPA with IgG4-RD overlap syndrome (97), activating neutrophils and stimulating the release of ROS (98, 99), further aggravating renal damage in MPA/GPA. In IgG4-RD, B cells also produce PDGF, which directly activates fibroblasts, promoting tissue fibrosis (100). This mechanism can exacerbate fibrosis in MPA/GPA-affected tissues in overlap syndromes.

While MPA does not feature granulomatous lesions (15), some individuals with MPA exhibit clinical features resembling IgG4-RD, including lymphadenopathy, elevated serum IgG4 levels, and TIN (9). For example, a patient with high serum IgG4, MPO-ANCA positivity, chest nodules, and elevated creatinine was diagnosed with IgG4 related kidney disease (IgG4-RKD) and MPA overlap syndrome based on renal biopsy findings (101).In some GPA cases, elevated serum IgG4 levels, fibrosis, vascular occlusion, and IgG4-positive plasma cell infiltration mimic IgG4-RD’s clinical histopathological features (102–104). Serum IgG4 concentrations correlate positively with organ involvement and predict disease recurrence (105, 106), so elevated IgG4 levels in MPA/GPA are associated with increased disease activity (107). The pathogenic role of IgG4 in IgG4-RD has not yet been clarified. Some studies suggest anti-inflammatory properties due to Fab arm exchange, poor C1q binding, and limited Fc receptor activation (108). However, elevated serum IgG4 may represent a failure of counter-regulation (1). Shiokawa et al. proved the pathogenic potential of IgG1/IgG4 antibodies from IgG4-RD causing pancreatic and salivary gland damage in a mouse model (109). Whether elevated IgG4 levels in MPA/GPA induce IgG4-RD progression requires further investigation. Notably, the number of plasmablasts is positively related to the levels of serum IgG4, inflammatory indicators, and the number of organs involved in IgG4-RD (110). Sometimes, IgG4-positive plasma cells were significantly increased in GPA biopsies of the sinuses, orbital/periorbital regions, kidneys, and dura mater (111). This suggests that IgG4-positive plasma cell infiltration in GPA may aggravate tumor-like proliferation and worsening IgG4-RD.

### 4.1 BAFF/APRIL

BAFF of the TNF family and APRIL, produced by innate immune cells, including neutrophils, monocytes, and macrophages, promote the survival and activation of B-cells (112). Increased production of BAFF and APRIL have been identified in several autoimmune diseases, such as systemic lupus erythematosus (SLE), AAV, rheumatoid arthritis (RA), and IgG4-RD (112, 113). They bind to B cell or memory B cell receptors, promoting activation, antibody production, and IgG class switching (114). In IgG4-RD, APRIL facilitates IgG4-positive plasma cell infiltration in affected tissues (47). Telitacicept, a BAFF/APRIL inhibitor, induces remission in refractory IgG4-RD, highlighting its critical role in disease pathogenesis (115). In AAV, BAFF/APRIL consistently elevated over normal patient’s matter at the active or remission stage (112). ANCAs stimulate neutrophils to produce BAFF, promoting autoreactive B cell activation and ANCA production (66). BAFF/APRIL levels correlate with MPA/GPA activity, and BAFF-driven autoreactive B-cell activation after B-cell depletion contributes to AAV relapse (116). Overall, BAFF and APRIL promote IgG4-positive plasma cell infiltration and IgG4/ANCA production, exacerbating IgG4-RD and MPA/GPA overlap syndromes.

## 5 The role of T cells in IgG4-RD and MPA/GPA overlap syndrome

T cells, key components of the adaptive immune system, contribute significantly to the pathogenesis of IgG4-RD and MPA/GPA by releasing pro-inflammatory cytokines and stimulating B cells to produce IgG4/ANCA antibodies (30, 117). Activated CD4+ T cells differentiate into various subsets, including Th1, Th2, Th17, regulatory T (Treg) cells, and follicular helper T (Tfh) cells, each playing distinct roles in these diseases.

### 5.1 Th1/Th2 cells

Th1 and Th2 cells are closely associated with the immune responses in IgG4-RD and AAV. Th1 cells, driven by IL-12, mediate cellular immunity, while Th2 cells, stimulated by IL-4, regulate humoral immunity (118, 119).Th1 cells are elevated individuals within IgG4-RD compared to controls, contributing to chronic inflammation and fibrosis through IFN-γ secretion (67). Increased Th1 cells correlate with IgG4-RD activity and IgG4 antibody levels (120), while IFN-γ induced Tfh proliferation further enhances IgG4 production (121). In GPA granulomas, Th1 cells are abundant, and IFN-γ secretion promotes granuloma formation (51), exacerbating GPA when IgG4-RD is present. IFN-γ levels also correlate with renal crescent formation and hyperplasia in MPA (68, 118), suggesting that IFN-γ-driven Th1 cells aggravate renal injury in IgG4-TIN and MPA/GPA overlap syndrome (122).

A previous peripheral CD4+ T cells in IgG4-associated dacryoadenitis and salivary gland inflammation revealed that the lacrimal and salivary glands are predominantly infiltrated by Th2 cells (123). Similarly, Th2-mediated immune-inflammatory response is predominant in IgG4-associated autoimmune pancreatitis and cholangitis (124), underscoring the significant role of Th2 cells in IgG4-RD pathogenesis (69).Th2 cells are also dominant in the nasal mucosa in Wegener’s granulomatosis (125).During the progression of GPA from localized granulomatosis to generalized vasculitis, a polarization shift from Th1 to Th2 responses occurs (126). This Th1 to Th2 conversion is a hallmark of GPA disease progression (126, 127). In case reports of IgG4-RD overlapping with MPA/GPA, the condition frequently manifests with systemic involvement (96), and ANCA-positive individuals with IgG4-RD are more likely to present with systemic symptoms (97). Therefore, Th2 cell polarization may dominate in overlap syndromes. Th2 cells secrete cytokines such as IL-4 and IL-13, which promote B-cell differentiation and IgG4-ANCA production (70). *In vitro* studies demonstrate that IL-4 stimulation alone induces the conversion of IgG to IgG4, significantly increasing plasma IgG4 concentrations (69), and IgG4 ANCA subtypes in individuals with overlap syndrome (97). Therapeutically, the anti-IL-4 receptor monoclonal antibody dupilumab has been shown to reduce tissue swelling in IgG4-RD and lower glucocorticoids requirements in affected individuals (71). These findings suggest that targeting IL-4 may serve as a common therapeutic strategy for both IgG4-RD and MPA/GPA overlap syndromes.

### 5.2 Th17/Treg cells

Th17 and Treg cells, which differentiate from CD4+ T cells, are central to autoimmune diseases, including IgG4-RD and MPA/GPA (128). Pro-inflammatory cytokines, such as IL-6, IL-23, and TGF-β, drive Th17 differentiations, while TGF-β alone favors Treg development (129). Both can be converted into each other.

Th17 cells, which produce IL-17, aggregate in IgG4-RD and MPA/GPA, promoting inflammation and fibrosis in affected tissues (72–74). They are also involved in a variety of autoimmune diseases, such as inflammatory bowel disease, AAV, SLE, and RA (128, 130, 131). IL-17 produced by Th17 cells contributes to fibrosis in IgG4-RD-affected tissues (132). Similarly, high levels of IL-17 in MPA/GPA may exacerbate IgG4-RD fibrosis. In MPA/GPA, Th17 cells play a central role by activating neutrophils and macrophages through IL-17 production (133). IL-17 enhances neutrophil expression of PR3 and MPO antigens, induces CXC chemokine release, and promotes adhesion molecule expression, facilitating neutrophil recruitment to inflammatory sites (75). Furthermore, IL-17 induces macrophages to secrete pro-inflammatory cytokines such as IL-1β and TNF-α, amplifying the inflammatory response (134). MPO-ANCA has been shown to stimulate IL-17 production, driving autoimmune anti-myeloperoxidase glomerulonephritis (74). Therefore, an increased Th17 and IL-17 in individuals with IgG4-RD may exacerbate ANCA-associated glomerulonephritis by activating neutrophils.

Treg cells are essential immunosuppressive cells that regulate inflammation by secreting TGF-β and IL-10 to inhibit pro-inflammatory cytokines production by macrophages and T cells (135).In IgG4-RKD, Tregs are significantly elevated, promoting IgG4 production by reducing IL-4 to IgE conversion, primarily via IL-10 secretion (76). Miyoshi et al. demonstrated that Treg levels positively correlate with serum IgG4 concentrations in IgG4-associated autoimmune pancreatitis (136). In IgG4-RKD, Tregs infiltrate renal tissue, promoting interstitial fibrosis by producing TGF-β (80). In MPA/GPA, Treg numbers increase during remission periods, suggesting a potential role in disease modulation (137). One study proposed that Tregs in MPA/GPA may differentiate from Th17 cells (138). However, in the presence of pro-inflammatory cytokines like IL-6, IL-23, and TGF-β, Tregs can convert into Th17 cells (81), perpetuating chronic autoimmune inflammation in MPA/GPA (139). This conversion may exacerbate the vascular inflammatory response in overlap syndromes involving IgG4-RD and MPA/GPA.

### 5.3 Tfh cells

Tfh cells are specialized CD4+ T cells involved in antibody class switching, plasma cell differentiation, and germinal center formation (140). These cells play pivotal roles in autoimmune diseases such as SLE, RA, IgG4-RD, AAV, and Sjögren’s disease (141). In IgG4-RD, Tfh cells proliferated, with a predominance of Tfh2 cells. Elevated Tfh2 levels correlate with IgG4-RD activity and serum IgG4 concentrations (142, 143), secreting IL-4 and IL-21 to promote IgG4 antibody production and B-cell proliferation (77, 144, 144). Tfh1 cells were also increased and positively correlated with IgG4-RD activity, independent of IgG4 levels (144).In MPA/GPA, Tfh2 cells increased significantly, promoting B cell proliferation, differentiation, and germinal center formation by secreting IL-4 and IL-21 (78, 79). Therefore, Tfh aggravates B cell proliferation and promotes the production of more IgG4-ANCA in IgG4-RD and AAV overlap syndromes. IL-21 produced by Tfh2 correlates with AAV activity and is identified as a risk factor for AAV activity (145), so high levels of IL-21 in IgG4-RD worsen AAV. Additionally, IL-21 assists IL-23 and TGF-β in Th17 differentiation (81), suggesting that elevated IL-21 in IgG4-RD contributes to Th17 polarization and overlap syndrome progression.

## 6 The role of macrophages in IgG4-RD and MPA/GPA overlap syndrome

Monocytes, as part of innate immunity, play critical roles in defending against pathogens, phagocytosing apoptotic cells, producing ROS, and presenting antigens (146).In IgG4-RD, monocytes secrete TGF-β and IL-1β, promoting fibrosis in affected tissues (147). In AAV, ANCAs activate monocytes to produce pro-inflammatory cytokines such as IL-1β, TNF-α, and IL-6, which, in turn, activate neutrophils (148) and contribute to tubulointerstitial injury (149). In IgG4-RD, IL-1β also produced by CD4+ CTLs, high IL-1β may exacerbate MPA/GPA by activating neutrophils. When peripheral blood mononuclear cells are stimulated with PR3 or MPO, it results in an elevated production of IL-6 (150), This heightened IL-6 level subsequently promotes fibroblast proliferation as well as the synthesis of collagen and fibronectin, thereby worsening fibrosis in the tissues affected by IgG4-RD (82). Additionally, IL-6 can stimulate the production of Tfh differentiation factors and B cell activating factors in IgG4-RD, thereby promoting Tfh cell differentiation and B cell antibody production (82). In overlap syndromes involving IgG4-RD and MPA/GPA, monocyte proliferation releases various inflammatory cytokines, promoting vasculitis and fibrosis in affected tissues.

Monocytes differentiate into macrophages in inflamed tissues, which can be polarized into two subtypes: M1 macrophages (classically activated) and M2 macrophages (alternatively activated) (151). M1/M2 polarization mirrors Th1/Th2 differentiation (152). Th1 cytokines, such as IFN-γ, drive M1 polarization, and M1 macrophages secrete IL-6, IL-12, and IL-23, which promote Th1 and Th17 cell differentiation. In contrast, Th2 cytokines, such as IL-4 and IL-13, induce M2 polarization, and macrophages secrete IL-33 to enhance Th2 differentiation (44, 152).

In IgG4-RD, M2 macrophages promote fibrosis by producing IL-33, TGF-β, and CCL-18, which upregulate collagen production by fibroblasts (35, 153). Serum levels of these cytokines correlate with fibrosis severity in IgG4-RD (154). IL-33 interacts with ST2 on Treg cells, inducing TGF-β production and promoting fibrosis in IgG4-RD tissues (155, 156), which promotes fibrosis of the tissues involved in IgG4-RD (87). In MPA/GPA, elevated IL-33 enhances Th2 cell activity, stimulating plasma cell differentiation and ANCA production (88, 157). In conclusion, IL-33 promotes plasma cell differentiation and IgG4 subtype ANCAs production in IgG4-RD with MPA/GPA overlap syndromes. MPO-ANCA contributes to M2 polarization, secreting more TGF-β and exacerbating fibrosis (62, 158). So, MPO-ANCA may worsen the fibrosis of the affected tissues in patients with IgG4-RD.

Both M1 and M2 macrophages are present in GPA granuloma, and their differentiation depends on specific cytokine settings (65). However, a study of nasal mucosal biopsies in GPA indicated predominant M2 polarization (60). In MPA/GPA, M2 macrophages infiltrate renal tissues, activating endothelial cells and myofibroblasts to secrete pro-fibrotic factors such as IL-33, CCL-18, and TGF-β (64). Notably, excessive infiltration of M2 macrophages correlates positively with elevated serum creatinine levels and an increased risk of end-stage renal disease in patients with AAV (159). Therefore, M2 macrophage accumulation worsens fibrosis in MPA/GPA-affected tissues when it overlaps with IgG4-RD. While M2 macrophages play an anti-inflammatory role by destroying apoptotic cells called efferocytosis (160). PR3 antigen expressed on activated neutrophils interacts directly with the “eat-me” signaling calpain on neutrophils, thereby impairing efferocytosis (161), resulting in incomplete clearance of neutrophils, T cells, and B cells, driving continued ANCA production and promoting granuloma formation. Thus, PR3 exacerbates tissue damage in IgG4-RD by impairing M2 macrophage efferocytosis (18).

## 7 Conclusion

IgG4-RD is a fibroinflammatory disease of unknown etiology with multi-system involvement that frequently overlaps with ANCA-associated vasculitis, posing significant diagnostic challenges. The pathogenesis of IgG4-RD and MPA/GPA involves complex interactions between B cells, T cells, and monocyte-derived macrophages, which proliferate, differentiate, and secrete cytokines that drive inflammation and fibrosis. These immune mechanisms not only contribute to disease progression but also highlight potential targets for therapeutic intervention. Understanding the interplay between these cells and cytokines provides valuable insights into the management of IgG4-RD and MPA/GPA overlap syndromes.
