# Supplementary material for: Immune cell crosstalk between ANCA-associated vasculitis and IgG4-related disease: an unresolved pathogenic link
Source: Front Immunol. 2025 Oct 16;16:1660956. doi: 10.3389/fimmu.2025.1660956 (PMC12571744; doi:10.3389/fimmu.2025.1660956)
Supplement: Supplementary file 1 [file Table1.docx]

|  | Study type | Clinical findings | Organ involvement | Serology | Imaging | Biopsy site and findings | Treatment | Outcome |
| --- | --- | --- | --- | --- | --- | --- | --- | --- |
| MPA and IgG4-RD overlap syndromes | Case(1) | Weakness,  weight loss,  purpura like rash on both lower extremities, numbness in both fingers,  cough,  expectoration,  anemia | Kidney (TIN, GN), Lung,  Pancreas,  Bile duct,  Mediastinum,  Axillary lymph nodes, Parotid gland. | p-ANCA+,  MPO-ANCA > 200 RU/mL,  IgG4: 17.16 g/L,  IgG: 35.1 g/L | Chest CT: enlarged multiple lymph nodes were present in the mediastinum and both axillae; Abdominal ultrasonography: thickening of the common bile duct wall, enlargement of both kidneys; Abdominal CT: enlarged pancreas. | Kidney biopsy: “pauci-immune” crescentic GN, massive IgG4+ PC infiltration in the renal (IgG4+/IgG+ PC ratio > 40%, and > 10 IgG4+ PC/HPF), TIN | MP (60 mg/day for 10 days), followed by oral prednisone (60 mg/day for 20 days with gradual tapering). | Gradual remission |
|  | Case(2) | Malaise and joint pain | Kidney (GN, TIN) | ANCA+,  MPO-ANCA: 55 IU/mL,  Scr: 227 µmol/L, IgG4: 1.94 g/L | Normal | Kidney biopsy：cellular and fibrous crescents with negative immunohistochemistry, TIN with a predominance of IgG4+ PC. | IV CYC 1 g and prednisolone 30 mg, then RTX. | Improved after the first dose of RTX, the second dose is planned. |
|  | Case(3) | Cough,  difficulty breathing, hemoptysis,  epistaxis | Nose,  Lung,  Kidney | p-ANCA+,  MPO-ANCA: 33 IU/mL,  IgG: 934 mg/dL, IgG4: 38 mg/dL | Chest, cranial, and upper airway CT: alveolar consolidation, with thickening of left maxillary sinus mucosa | Nasal biopsy: neutrophil infiltration,  fibrinoid degeneration. Specific immunohistochemical staining：25 IgG4+ PC/HPF | CYC and prednisolone (70mg/kg/day), Plasmapheresis, Hemodialysis. | Hemodialysis,  Renal transplant |
|  | Case(4) | Cough,  hemoptysis,  loss of appetite,  fatigue,  hearing loss | Lung,  Kidney,  Ear,  Sinuses | p-ANCA+,  MPO-ANCA: 275.27 U/mL,  PR3-ANCA: 2.181 U/mL,  IgG:19.7g/L,  IgG4: 4.07 g/L, IgG4/IgG: 20.7%, Scr: 481 µmol/L | CT: bilateral otitis media with mastoiditis and bilateral ethmoid sinusitis; Chest CT: Right maxillary sinusitis, multiple subpleural small nodules, inflammation in the upper lobe of the right lung, ground-glass opacities in the lower lobes of both lungs | Kidney biopsy：cellular crescents,  fibrotic crescents, multifocal inflammatory cell infiltration in the tubulointerstitial area, IgG4+ PC > 10/HPF, IgG4+PC/IgG PC > 40%. | IV MP (pulse 0.5g/d for 3 days)，then IV 40 mg/d  IV CYC，0.2-0.4 g，biweekly | Gradual remission |
|  | Case(5) | Chronic cough, dysphonia,  dysphagia,  hypoacusis,  fever,  weight loss,  mild anemia,  weakness | Lung,  Kidney (GN, TIN), Lymph nodes,  PNS,  Mastoiditis | Scr: 2.69 mg/dL, MPO-ANCA: 84%AU,  IgG4: 529mg/dL, IgG4/IgG: 27% | PET/CT: moderate18F-FDG uptake of mediastinic, parathracheal, ilar, and axillary lymph nodes, Electroneurography and electromyography： neurogenic damage with signs of denervation,  MRI: Mastoiditis | 2 years ago: Gallbladder: LP and eosinophilic infiltrate, fibrosis, IgG4+/IgG+ PC ratio > 40%, and 45 IgG4+ PC/HPF  Kidney biopsy: Renal interstitial intense lymphomonocytic infiltration, with IgG4+ PC and eosinophils. Active inflammation and atrophy of some renal tubules | IV prednisone (0.5 g/d for 3 days) followed by IV CYC (1 g/month, 7g total dose) | Remission |
|  | Retrospective two-center study (6) | NA | Kidney (GN, TIN), Lung | MPO-ANCA+, IgG4: 2.07g/L | NA | Kidney biopsy: cellular crescent, 17 IgG4+ PC/HPF | NA | NA |
|  |  | NA | Kidney (GN, TIN),  PNS | MPO-ANCA+, IgG4c: 8.35g/L | NA | Kidney biopsy: cellular crescent,  fibrous crescent, 29 IgG4^+^ PC/HPF, Storiform fibrosis. | NA | NA |
|  |  | NA | Kidney (GN, TIN), Lung, Eye, Lymph nodes | MPO-ANCA+, IgG4: 5.55 g/L | NA | Kidney biopsy: cellular crescent, 100 IgG4+ PC/HPF. | NA | NA |
|  |  | NA | Kidney (GN, TIN) | MPO-ANCA+, IgG4: 1.26g/L | NA | Kidney biopsy: cellular crescent, fibrous crescent, 54 IgG4+ PC/HPF | NA | NA |
|  |  | NA | Kidney (GN, TIN),  PNS | MPO-ANCA+, IgG4: 2.47g/L | NA | Kidney biopsy: cellular crescent, 50 IgG4+ PC/HPF | NA | NA |
|  |  | NA | Kidney (GN, TIN) | PR3-ANCA+,  IgG4: 1.86g/L | NA | Kidney biopsy: cellular crescent, 47 IgG4+ PC/HPF, Storiform fibrosis | NA | NA |
|  |  | NA | Kidney (GN, TIN), Lung, PNS, Eye | MPO-ANCA+, IgG4: 2.86g/L | NA | Kidney biopsy: cellular crescent, 100 IgG4+ PC/HPF | NA | NA |
|  |  | NA | Lung,  kidney (GN, TIN) | MPO-ANCA+, IgG4: 8.76g/L | NA | Kidney biopsy: cellular crescent, fibrous crescent, 47 IgG4+ PC/HPF | NA | NA |
|  |  | NA | Kidney (GN, TIN), lung, PNS | MPO-ANCA+, IgG4: 2.08g/L | NA | Kidney biopsy: cellular crescent, fibrinoid necrosis, 25 IgG4+ PC/HPF | NA | NA |
|  |  | NA | Kidney (GN, TIN), lung, eye, PNS | MPO-ANCA+, IgG4: 7.62g/L | NA | Kidney biopsy: cellular crescent,  fibrinoid necrosis, 104 IgG4+ PC/HPF | NA | NA |
|  |  | NA | Kidney (GN, TIN) | MPO-ANCA+, IgG4: 6.42g/L | NA | Kidney biopsy: cellular crescent, 5 fibrous crescent, 50 IgG4+ PC/HPF | NA | NA |
|  |  | NA | Kidney (GN, TIN), lung, PNS | MPO-ANCA+, IgG4: 6.52g/L | NA | Kidney biopsy: cellular crescent, 1 fibrous crescent, 40 IgG4+ PC/HPF | NA | NA |
|  |  | NA | Kidney (GN, TIN) | MPO-ANCA+, IgG4: 3.83g/L | NA | Kidney biopsy: cellular crescent, 20 IgG4^+^ PC/HPF | NA | NA |
|  |  | NA | Kidney (GN, TIN),  lung | MPO-ANCA+, IgG4: 2.30g/L | NA | Kidney biopsy: 2 cellular crescent, fibrous crescent, >10 IgG4+ PC/HPF | NA | HD |
|  |  | NA | Kidney (GN, TIN) | MPO-ANCA+, IgG4: 5.84g/L | NA | Kidney biopsy: cellular crescent, >10 IgG4^+^ PC/HPF | NA | HD |
|  | Case(7) | nausea,  poor appetite,  nerve deafness,  weight loss | Kidney (TIN, GN),  Ear | MPO-ANCA: 168 IU/ mL,  IgG4: 7.32 g/L,  IgG: 17.23 g/L,  Scr: 413µmol/L | NA | Kidney biopsy: cellular crescents and fibrous crescents, IgG and fibrinogen-related antigen deposition in mesangial and capillary wall. Diffuse infiltration of mononuclear cells, PC, and eosinophilic cells in the tubulointerstitium. SF, > 10 IgG4+ PC/HPF | IV MP pulse (0.5 g/day for 3 days), oral prednisone (40 mg/ day with gradual tapering).  IV CYC was given simultaneously (50 mg/day, 9 g total dose) | Partial recovery |
|  |  | Weakness,  fever,  weight loss | Kidney (TIN, GN) | MPO-ANCA: 95.3I U/mL,  IgG4: 4.26 g/L,  IgG: 32.47 g/L,  Scr: 274.4µmol/L | NA | Kidney biopsy: cellular crescents, large fibro-cellular crescents, large fibrous crescents，fibrinoid necrosis in some small arteries. Diffuse infiltration of mononuclear cells, PC, and eosinophils in the tubulointerstitium. SF, > 10 IgG4+ PC/HPF, pauci-immune deposition | IV MP pulse (0.5 g/day for 3 days), then: oral prednisone (50 mg/ day) and CYC simultaneously (50 mg/day) | Partial recovery |
|  | Sigle-center study retrospective (8) | Fever, Dacryoadenitis, interstitial lung disease, arthritis | Kidney (GN),  Lung,  ENT,  Joint | MPO-ANCA+ | NA | Lacrimal gland biopsy: NA | GC, renal replacement therapy | No remission |
|  | Case(9) | enlarged kidney cysts, AKI | Kidney (GN, TIN), artery | IgG: 2453 mg/dL, IgG4: 378mg/dL, MPO-ANCA: 280 IU/mL | CT: multiple low-density lesions of the bilateral kidneys and hypertrophic soft tissue around the right common iliac artery | Kidney biopsy: segmental tuft necrotic glomeruli, infiltration of PC in the tubulointerstitium,  IgG4+ PCs > 50/HPF | IV high dose GC and CYC | Remission |
|  | Retrospective multicenter observational study(10) | NA | LN(TIN),  Pancreas (AIP) | NA | NA | Kidney biopsy：Fibrosis, Lympho-plasmocytic infiltrate, Neutrophilic infiltrate, IgG4+ PC/HPF > 10, IgG4+/IgG+ PC ratio 40% | NA | NA |
| MPA and IgG4-RD overlap syndromes | case(11) | pain,  weight loss,  cough,  epistaxis,  bilateral conjunctival hemorrhage,  hearing loss | Ear,  kidney,  sclera,  lung,  kidney,  pancreas, c  lymph,  ENT | ANCA +++,  PR3-ANCA: 200U, IgG4: 565mg/dL | Chest and abdominal CT：pulmonary nodules Swollen axillary lymph nodes, pancreatic swelling.  Endoscopic ultrasound:  overall thickening of the pancreas with edematous changes beside the aorta, and adjacent to the inferior vena cava. | Adipose tissue surrounding the parathyroid gland: IgG4+PC/IgG+ > 50% | IV MP (1 g daily for 3 days),  cIV CYC (1 g monthly for 6 months), with MP at 1 mg/kg/day. | Remission |
|  | Case(12) | Weight loss,  fever,  night sweats | Nose,  Kidney (TIN, GN), aorta | ANCA+,  MPO-ANCA 9U/L, IgG4 1.85 g/L,  Scr 94µmol/L | Chest CT: concentric thickening of the ascending aorta  Abdominal MRI：Edema of the media of the abdominal aorta  18-FDG：Significant increase in FDG uptake in the ascending aorta, brachiocephalic trunk, abdominal aorta and kidneys. | Kidney biopsy: multiple small areas of necrosis with cellular debris and focal giant cell infiltration, crescent formation and segmental fibrinoid necrosis, negative pauci- immune**,**  TIN, 23 IgG4+ PC/HPF, IgG4+PC/IgG+ PC 50% | IV MP pulse (0.5 g/day 3 days),  oral prednisone (1 mg/kg/day).  IV RTX with 2 pulses of 1 g at intervals of 2 weeks, followed IV 500 mg rituximab at 6 months intervals. | Remission |
|  | Case(13) | Fatigue,  weight loss,  dry cough,  left eye vision loss, muscle pain and weakness | Kidney (GN, TIN), peripheral nerve system  aortitis | MPO–NCA+,  C-ANCA: 1:320, MPO-ANCA: 42.3 U/mL,  IgG: 2739mg/dL, IgG4: 965 mg/dL | High-resolution chest CT: concentric thickening of the ascending aorta and aortic arch, extending to the left subclavian artery,  18F-FDG PET-CT: thickening of the aortic arch wall with abnormally low metabolism, hypermetabolism in the bilateral renal cortices, and diffuse increased metabolism in the bone marrow.  Lower extremity nerve conduction studies: a length-dependent  motor-sensory axonal polyneuropathy. | Kidney biopsy: TIN, pauci-immune GN with crescent formation and granulomas, obliterative endarteritis, and PC infiltration, 40 IgG4+ PC/HPF, 44 IgG+ PC/HPF, IgG4/IgG ratio 90% | Oral prednisone 1 mg/kg/day for 1 month with subsequent tapering and IV monthly CYC (500 mg/m2 body surface area)  biweekly | Remission,  Dialysis-free |
|  | Case(14) | Multiple ulcers on the face and scalp | Eyes,  mucous membranes, skin | IgG4 537mg/dL,  ANCA negative | Orbital and paranasal sinus CT scan: orbital ellulitis, dacryoadenitis, right ethmoid sinusitis. | Skin biopsy: dense infiltration by lymphocytes and plasma cells, with  arteritis. 200–250 IgG4+ PC/HPF, IgG4+/IgG+ 30% | 20 mg prednisolone,  chloroquine  250 mg/day  colchicine0.6mg/day | Improvement within 8 weeks |
|  | Case(15) | Abdominal pain,  joint pain,  bloody diarrhea,  weight loss,  purpura on both lower extremities,  bilateral conjunctival hyperemia,  oral ulcers, and swelling of the right parotid gland. | pancreas,  parotid gland,  ENT,  skin,  joints,  kidney | c-ANCA+,  PR3-ANCA > 300 U,  IgG4: 5.34 g/L | Abdominal ultrasound: a mass in the pancreatic region,  Chest ultrasound: pleural effusion  Cervical MRI: swelling of the right parotid gland | Pancreas biopsy：SF, PC infiltration, obliterative phlebitis, IgG4+/IgG PC>40%, >10 IgG4+ PC /HPF  Kidney biopsy：cellular or fibro-cellular crescents, Tubulointerstitial active inflammation rich in eosinophils and PC was prominent | induction：IV MP (pulse 20 mg/kg for 3 days);  IV MP (pulse continued for 5 days)  IV CYC (500 mg/m2/dose)  maintenance：oral prednisolone (10 mg/day)  IV RTX (375 mg/m2 once weekly for 4 weeks | Remission |
|  | Case(16) | Difficulty in urination, pelvic discomfort, low-grade fever, bilateral hearing loss, nasal congestion, nasal bleeding | ENT,  Kidney（TIN, GN） | IgG4: 112 mg/dL, PR3-ANCA+ | Nasal endoscopy: presence of nasal polyps, nasal crusting, and posterior nasal discharge.  Paranasal sinus CT: submucosal thickening of the maxillary sinuses, with partial involvement of the ethmoid and sphenoid sinuses. | Right kidney biopsy: necrotizing crescentic GN;  Left kidney biopsy: SF, and IgG4/IgG ratio>40%;  Nasal mucosa biopsy: IgG4/IgG ratio of 40% with lymphocytic vasculitis of small arteries. | Induction therapy：  IV prednisolone  (1 mg/kg),  IV RTX 1.0g twice.  Maintenance therapy: IV RTX (500mg rituximab every 6 months for the first 2 years and every 9 months thereafter). | Remission |
|  | Case (17) | Fever,  headache,  earache and hearing loss | ENT findings,  Lung,  Kidney (GN, TIN) | IgG: 1726 mg/dL,  IgG4:407.0mg/dL, MPO/PR3-ANCA negative | Head CT: right maxillary sinusitis.  Chest CT: infiltrates in the upper and middle lobes of both lungs. | Lung biopsy: granuloma and necrotizing vasculitis;  Renal biopsy: TIN, IgG4+/IgG+ cell ratio was about 50% and IgG4+ cells ≥ 10/HPF. | IV MP pulse for 3 days  Oral prednisolone（1 mg/kg/day）and IV CYC 500 mg monthly | Remission |
|  | Case (18) | Sputum production, hemoptysis, myalgia, arthralgia, fatigue, fever, chills, and diffuse tender cervical lymphadenopathy. | Kidney（GN）,  lung | IgG4: 289 mg/dL, PR3-ANCA+ | Chest CT: Diffuse alveolar hemorrhage and enlargement of the mass in the left upper lobe of the lung. Bronchoalveolar lavage: Bronchoscopy revealed significant hemorrhage in the right main bronchus and right middle lobe. | Renal biopsy: diffuse necrotizing, crescentic GN  Lung biopsy: IgG4+ cells >30/HPF,  IgG4/IgG ratio >10% | IV MP（pulse 1g/day for 3 days）  IV CYC pulse monthly  seven cycles of plasma pheresis,  Then AZA as maintenance therapy for GPA | Remission  succumbed to the small cell lung cancer 1 year after this admission |
|  | Case (19) | Fever, weight loss, hoarseness, bilateral dacryoadenitis, and nasal septum ulcer | ENT,  Lung | MPO-ANCA+: (ELISA 3.4 UA)  IgG4: 2.27 g/L | Chest CT: Consolidation in the upper lobes of both lungs and enlargement of multiple mediastinal lymph nodes.  Nasal laryngoscopy: granulomas on both vocal cords. | Lung biopsy: spoke-wheel fibrosis accompanied by granulation tissue formation, with extensive infiltration of PC and some lymphocytes. IgG4+ PC > 20/HPF | IV MP 500 mg for 3 days,  oral prednisone gradual tapering | Remission |
|  | Case (20) | Left lacrimal gland enlargement,  proptosis,  redness, and anterior scleritis | Orbital and lung, Lacrimal gland | IgG4: 253mg/dl, PR3-ANCA: 423AU | Gadolinium-enhanced brain and orbital MRI: Enlargement of the left lacrimal gland, surrounded by inflammatory tissue adjacent to the lateral rectus and superior rectus muscles.  PET/CT: Demonstrated significantly increased uptake of 18F-FDG in above inflammatory tissue.  Chest CT: confirmed the presence of two round nodules and one cavitary mass in the right lung. | Lung biopsy: chronic granulomatous inflammation, leukocytoclastic vasculitis, and areas of geographic necrosis and numerous IgG4+ PC；  Lacrimal gland biopsy: SF with abundant IgG4+ PC (IgG4+/IgG+ PC ratio > 40%) | Oral 1 mg/kg prednisone,  IV RTX 1.0g twice（15 days apart） | Remission |
|  | Case (21) | Amenorrhea with bilateral galactorrhea, polydipsia, polyuria, temporal headache, blurred vision, worsening proptosis, and purpura | Lung,  Skin,  Eyes,  pituitary | IgG4: 377mg/dl, MPO-ANCA+, IgG4/IgG > 50% | Head MRI: diffuse enlargement of the pituitary gland with loss of the posterior pituitary hyperintense signal.  Orbital MRI: bilateral orbital pseudotumor.  Chest CT: focal ground-glass opacity in the right middle lobe and multiple pulmonary nodules. | Skin biopsy: leukocytoclastic vasculitis  Orbital biopsy ：confirmed the presence of IgG4-RD | MP, AZA, RTX  Improved with RTX | Improved  with RTX |
|  | Case(22) | Arthritis,  sinusitis,  fever,  skin rash,  weight loss | Kidney,  ENT,  skin,  Retroperitoneal tissue | PR3-ANCA+,  PR3-ANCA > 200 U/L,  IgG4 normal | Ultrasound: Hydronephrosis  PET-CT: A 3.7 × 7.4 cm retroperitoneal tissue mass adjacent to the abdominal aorta.  Several subtle infiltrative lesions with increased FDG uptake are noted in the right lung. | Nasal biopsy: PC infiltration, scattered lymphocytes, and eosinophils  Retroperitoneal fibrosis biopsy: focal storiform configuration, IgG4+ cells 30/HPF | Prednisolone | Reduction of  the retroperitoneal  mass after  1 year |
|  | Retrospective two-center study (6) | NA | Kidney (GN, TIN), Lung,  AIP,  ENT | MPO-ANCA+,  IgG4 4.06 g/L  Scr: 512µmol/L | NA | Kidney biopsy: 25 normal glomeruli, 5 cellular crescent, 2 Fibrous crescent, 130 IgG4+ PC/HPF，SF | NA | NA |
|  |  | NA | Kidney (TIN,GN）  ENT,  GT,  PNS | MPO-ANCA+, IgG4: 2.13 g/L,  Scr: 792µmol/L | NA | Kidney biopsy: 6 normal glomeruli, 11 cellular crescent, 0 Fibrous crescent, 33 IgG4+ PC/HPF | NA | NA |
|  |  | NA | Kidney (GN, TIN） ENT,  Eye | Scr: 184µmol/L, MPO-ANCA+, IgG4: 5.33 g/L | NA | Kidney biopsy: 15 normal glomeruli, 26 cellular crescent, 3 Fibrous crescent, 200 IgG4+ PC/HPF | NA | HD |
|  | Retrospective multicenter observational study (10) | TIN | kidney | NA | NA | Kidney biopsy: Fibrosis, lymphoplasmacytic infiltrate, Neutrophilic infiltrate, IgG4+ PC/HPF > 30, IgG4+/IgG+ PC ratio 40% | NA | NA |
|  |  | Renal mass | Kidney, Pancreas | NA | NA | Kidney biopsy: Fibrosis, Lympho-plasmocytic infiltrate, IgG4+/IgG+ PC ratio 50%, Eosinophilic infiltrate | NA | NA |
|  |  | TIN  Thyroiditis | Kidney,  thyroid | NA | NA | Kidney biopsy: Fibrosis, Lympho-plasmocytic infiltrate, IgG4+ PC/HPF > 10 | NA | NA |
|  |  | Orbital mass, EAF (eosinophilic angiocentric fibrosis) | Orbital | NA | NA | Orbital mass: Fibrosis, Lympho-plasmocytic infiltrate, Eosinophilic infiltrate, IgG4+/IgG+ PC ratio 40%, Neutrophilic infiltrate, Vasculitis, Granuloma, Necrosis | NA | NA |
|  |  | Orbital mass, Mediastinal fibrosis | Orbital Mediastinal | NA | NA | Orbital mass: Lympho-plasmocytic infiltrate, Eosinophilic infiltrate, IgG4+ PC/HPF > 10 | NA | NA |
|  |  | Orbital mass, Sinusitis | Orbital,  Nose | NA | NA | ENT biopsy：Lympho-plasmocytic infiltrate, Neutrophilic infiltrate, IgG4+ PC/HPF > 60, Vasculitis, Granuloma | NA | NA |
|  |  | Myocardial Periaortitis | heart | NA | NA | Heart biopsy: Fibrosis, Lympho-plasmocytic infiltrate, Eosinophilic infiltrate, IgG4+ PC/HPF > 50, Neutrophilic infiltrate | NA | NA |
|  |  | Periaortitis | Aorta | NA | NA | Aorta biopsy: Obliterative phlebitis, Fibrosis, Lympho-plasmocytic infiltrate, Neutrophilic infiltrate, 50 IgG4+ PC/HPF, IgG4+/IgG+ PC ratio > 40% | NA | NA |
|  |  | Mediastinal fibrosis | Mediastinum | NA | NA | Mediastinum biopsy: Fibrosis, 13 IgG4+ PC/HPF, IgG4+/IgG+ PC ratio 50% | NA | NA |

AAV, ANCA-associated vasculitis; ANCA, Antineutrophil cytoplasmic antibody; ANCA-GN: antineutrophil cytoplasmic antibody-associated glomerulonephritis;

AZA，Azathioprine；CT, Computed Tomography; CYC, Cyclophosphamide; eGFR, Estimated glomerular filtration rate; ENT, Ear, nose and throat; FDG, Fluorodeoxyglucose; GC, Glucocorticoid; GN, Glomerulonephritis; GPA, Granulomatosis with polyangiitis; GT, Gastrointestinal tract; HPF, High power field; HD, Hemodialysis; IgG4-RD, IgG4-related disease; IV, Intravenous; MP, Methylprednisolone; MPA, Microscopic polyangiitis; MPO-ANCA, Myeloperoxidase-antineutrophil cytoplasmic antibody; MRI, Magnetic Resonance Imaging; NA, not available; PC, plasma cells; PET-CT, Positron Emission Tomography-Computed Tomography; PNS, Peripheral nervous system; PR3-ANCA, Proteinase 3-antineutrophil cytoplasmic antibody; RTX, Rituximab; Scr, Serum creatinine; SF, storiform fibrosis ; TIN, tubulointerstitial nephritis.

1. He R, Ma M, Luo P, Guo Q. An overlap of IgG4-related tubulointerstitial nephritis and microscopic polyangiitis-associated glomerulonephritis: a case-based review. *Clin Rheumatol* (2023) 42:1459-67. doi: 10.1007/s10067-022-06493-5

2. Wu HHL, Wang CCY, Woywodt A, Ponnusamy A. Concurrent presentation of IgG4-related tubulointerstitial nephritis and ANCA MPO crescentic glomerulonephritis. *Clin Nephrol Case Stud* (2022) 10:47-53. doi: 10.5414/cncs110852

3. Torres Tienza S, Rueda Correa F, Campos Téllez S, Jareño Esteban JJ. ANCA-Associated Vasculitis Presenting With Alveolar Hemorrhage and Renal Involvement and IgG4-Related Disease: A New Overlap Syndrome. *Arch Bronconeumol* (2022) 58:431-32. doi: 10.1016/j.arbres.2022.02.017

4. Liang P, Chen W, Yue S, Han Q, Zhu L, Li J, et al. An overlap of antineutrophil cytoplasmic antibody-associated vasculitis and IgG4-related disease: distinct clinicopathologic clues for precise diagnosis. (2021). doi: 10.21203/rs.3.rs-161443/v1

5. Capecchi R, Giannese D, Moriconi D, Bonadio AG, Pratesi F, Croia C, et al. Renal Involvement in IgG4-Related Disease: From Sunlight to Twilight. *Front Med (Lausanne)* (2021) 8:635706. doi: 10.3389/fmed.2021.635706

6. Li ZY, Wang X, Xia X, Yu XJ, Wang SX, Chen W, et al. An overlap of antineutrophil cytoplasmic antibody (ANCA)-associated glomerulonephritis and IgG4-related kidney disease. *Clin Chim Acta* (2020) 501:12-19. doi: 10.1016/j.cca.2019.11.030

7. Wang GQ, Chen YP, Cheng H, Xu XY, Sun LJ, Dong HR. Antineutrophil cytoplasmic antibody and/or antiglomerular basement membrane antibody associated crescentic glomerulonephritis in combination with IgG4-related tubulointerstitial nephritis. *Clin Exp Rheumatol* (2019) 37:279-85. doi:

8. Martín-Nares E, Zuñiga-Tamayo D, Hinojosa-Azaola A. Prevalence of overlap of antineutrophil cytoplasmic antibody associated vasculitis with systemic autoimmune diseases: an unrecognized example of poliautoimmunity. *Clin Rheumatol* (2019) 38:97-106. doi: 10.1007/s10067-018-4212-1

9. Watanabe T, Kanda M, Fukaya S, Ogawa Y, Akikawa K. Rapidly progressive glomerulonephritis caused by overlap syndrome of IgG4-related tubulointerstitial nephritis and myeloperoxidase-antineutrophil cytoplasmic antibody-associated necrotising glomerulonephritis. *Clin Exp Rheumatol* (2018) 36 Suppl 111:172-73. doi:

10. Danlos FX, Rossi GM, Blockmans D, Emmi G, Kronbichler A, Durupt S, et al. Antineutrophil cytoplasmic antibody-associated vasculitides and IgG4-related disease: A new overlap syndrome. *Autoimmun Rev* (2017) 16:1036-43. doi: 10.1016/j.autrev.2017.07.020

11. Korkmaz C, Yıldırım R, Dinler M, Cansu DU. Coexistence of IgG4-related disease and ANCA-associated vasculitis: case report and review of the literature. *Rheumatol Int* (2024) 44:557-72. doi: 10.1007/s00296-023-05419-x

12. Kuske L, Khalifa A, Wibisono A, Bräsen JH, Witte T. MPO-ANCA-positive granulomatosis with polyangiitis and concurrent IgG4-related disease with periaortitis and tubulointerstitial nephritis: A case report of a new overlap syndrome? *Int J Rheum Dis* (2023). doi: 10.1111/1756-185x.14680

13. Faz-Muñoz D, Hinojosa-Azaola A, Mejía-Vilet JM, Uribe-Uribe NO, Rull-Gabayet M, Muñoz-Castañeda WR, et al. ANCA-associated vasculitis and IgG4-related disease overlap syndrome: a case report and literature review. *Immunol Res* (2022) 70:550-59. doi: 10.1007/s12026-022-09279-8

14. Julanon N, Chularojanamontri L, Sitthinamsuwan P, Sukpanichnant S. Multiple facial ulcers as a presentation of localized granulomatosis with polyangiitis associated with IgG4-related disease. *Clin Exp Dermatol* (2021) 46:610-13. doi: 10.1111/ced.14503

15. Demir AM, Aydin F, Acar B, Kurt T, Poyraz A, Kiremitci S, et al. IgG4-related disease and ANCA positive vasculitis in childhood: a case-based review. *Clin Rheumatol* (2021) 40:3817-25. doi: 10.1007/s10067-021-05635-5

16. Boncoraglio MT, Prieto-González S, Fernandes-Serodio J, Corral-Molina JM, Solé M, Hernández-Rodríguez J. Simultaneous presentation of granulomatosis with polyangiitis (GPA) and immunoglobulin G4-related disease (IgG4-RD). Leaving an open question: widening the spectrum of a single disease or real overlap? *Mod Rheumatol Case Rep* (2021) 5:108-12. doi: 10.1080/24725625.2020.1782028

17. Kawashima H, Utsugi A, Shibamiya A, Iida K, Mimura N, Ohashi H, et al. Consideration concerning similarities and differences between ANCA-associated vasculitis and IgG-4-related diseases: case series and review of literature. *Immunol Res* (2019) 67:99-107. doi: 10.1007/s12026-019-9070-7

18. Abbass K, Krug H. Granulomatosis with polyangiitis in a patient with biopsy-proven IgG4-related pulmonary disease and coincident small cell lung cancer. *BMJ Case Rep* (2019) 12. doi: 10.1136/bcr-2018-226280

19. Bravais J, Pogliaghi M, Polivka M, Sène D, Roriz M. IgG4-related disease and ANCA positivity: an overlap syndrome? *Qjm* (2017) 110:749-50. doi: 10.1093/qjmed/hcx134

20. Della-Torre E, Lanzillotta M, Campochiaro C, Bozzalla E, Bozzolo E, Bandiera A, et al. Antineutrophil cytoplasmic antibody positivity in IgG4-related disease: A case report and review of the literature. *Medicine (Baltimore)* (2016) 95:e4633. doi: 10.1097/md.0000000000004633

21. Alexandraki KI, Kaltsatou M, Chatzellis E, Goules AV, Boutzios G, Kolomodi D, et al. Hypophysitis in IgG4-Related Disease Associated with p-ANCA Vasculitis. *Am J Med* (2016) 129:e25-7. doi: 10.1016/j.amjmed.2015.11.021

22. Lomborg N, Marcussen N, Junker P. Retroperitoneal fibrosis as the presenting manifestation of IgG4-related disease in a patient previously diagnosed with granulomatosis with polyangiitis (Wegener's): one disease or two? *Scand J Rheumatol* (2014) 43:345-7. doi: 10.3109/03009742.2014.905628
